# Thoracic Reconstruction Using a Poly-L-Lactic Acid Mesh Plate for an Infant with Pectus Excavatum and Complex Congenital Heart Disease Preventing Chest Closure

**DOI:** 10.70352/scrj.cr.25-0611

**Published:** 2025-11-26

**Authors:** Masaya Yamoto, Juma Obayashi, Hiroki Ito, Yu Sugai, Akiyoshi Nomura, Hiromu Miyake, Koji Fukumoto

**Affiliations:** 1 Department of Pediatric Surgery, Osaka City General Hospital, Osaka, Osaka, Japan; 2 Department of Surgery, Shizuoka Children’s Hospital, Shizuoka, Shizuoka, Japan; 3 Department of Cardiovascular Surgery, Shizuoka Children’s Hospital, Shizuoka, Shizuoka, Japan

**Keywords:** pectus excavatum, sternal compression, congenital heart disease, infant thoracic reconstruction, poly-L-lactic acid (PLLA) mesh plate

## Abstract

**INTRODUCTION:**

Pectus excavatum (PE) is the most common anterior chest wall deformity, but surgical intervention during infancy is rarely indicated. In patients with complex congenital heart disease (CHD), however, PE may severely compromise mediastinal capacity, particularly after repeated sternotomies. Although delayed sternal closure is a standard approach to address postoperative hemodynamic instability, in cases where chest wall deformity directly compresses the heart, definitive thoracic reconstruction may be required.

**CASE PRESENTATION:**

We report a 9-month-old boy with right atrial isomerism, single atrium, single ventricle, and total anomalous pulmonary venous connection (TAPVC). After initial TAPVC repair with pulmonary artery banding at 2 months of age, he developed progressive PE. At reoperation for pulmonary venous stenosis release and pulmonary artery re-banding, correction of mediastinal rotation returned the enlarged heart to the midline. Attempts at chest closure resulted in abrupt elevation of central venous pressure and systemic hypotension due to direct cardiac compression by the sternum and costal cartilages. Temporary skin-only closure was performed. Definitive thoracic reconstruction was undertaken 48 hours later using a modified Rehbein technique with a bioabsorbable poly-L-lactic acid (PLLA) mesh plate, molded into a convex shape and fixed anterior to the sternum. This approach increased mediastinal volume and allowed stable chest closure. Postoperatively, right phrenic nerve palsy required noninvasive ventilatory support for 3 weeks. The patient recovered and was discharged 2 months later in good condition.

**CONCLUSIONS:**

This case demonstrates that in infants with complex CHD, severe PE may render chest closure impossible, leading to life-threatening hemodynamic compromise. Thoracic reconstruction using an absorbable PLLA mesh plate provided temporary but effective external fixation, securing mediastinal space without impairing growth. This growth-sparing strategy may represent a valuable salvage option when conventional closure fails in pediatric cardiac surgery.

## Abbreviations

CHD
: congenital heart disease
CPAP
: continuous positive airway pressure
CVP
: central venous pressure
PE
: pectus excavatum
PLLA
: poly-L-lactic acid
TAPVC
: total anomalous pulmonary venous connection

## INTRODUCTION

PE is the most common congenital chest wall deformity, with an estimated prevalence of up to 1 in 300–400 live births.^1)^ Although many patients remain asymptomatic during childhood, severe cases can cause cardiopulmonary impairment, including right ventricular outflow obstruction and syncope,^2)^ alterations in ventricular–arterial coupling,^3)^ and even associations with mitral valve prolapse.^4)^ The impact of PE on pulmonary physiology has also been well documented, with deformity severity correlating with reduced lung volumes and exercise tolerance.^5)^ Surgical repair of PE has evolved substantially over the past decades. The Ravitch technique was historically the standard,^6)^ but since the introduction of the Nuss procedure, minimally invasive repair has become widely adopted, with favorable outcomes across different age groups.^7)^ However, the optimal timing of repair remains debated, particularly in infants and young children, where recurrence risk and growth-related concerns are significant.^1,8)^ In patients with CHD, PE presents unique challenges. Previous studies have highlighted the co-occurrence of chest wall deformities and CHD,^9)^ and simultaneous or staged surgical repair strategies have been reported.^10–12)^ For example, Takabayashi et al. described suture repair of PE at the time of infant cardiac surgery,^13)^ while Chen et al. and Li et al. reported outcomes of Nuss repair following CHD surgery.^10,14)^ Despite these reports, PE requiring intervention in infancy is exceedingly rare, and consensus on timing and methods is lacking.

We present the case of an infant with complex CHD in whom severe PE led to intraoperative failure of chest closure due to direct cardiac compression. Thoracic reconstruction was performed using a bioabsorbable PLLA mesh plate, providing temporary external fixation without compromising growth.

## CASE PRESENTATION

**Fig. 1:**
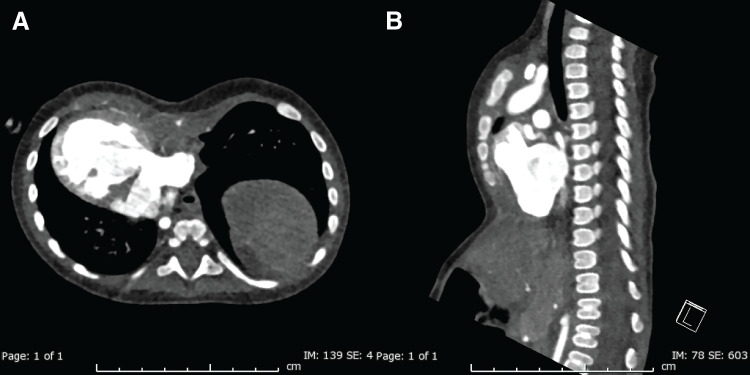
Preoperative contrast-enhanced CT. (**A**) Axial view demonstrating severe pectus excavatum with a Haller index of 3.5 and hypertrophic retrosternal soft tissue. (**B**) Sagittal view confirming the depth of sternal depression and reduced mediastinal space.

The patient was a 9-month-old boy with right atrial isomerism, single atrium, single ventricle, and TAPVC type IV. He had undergone primary TAPVC repair combined with main pulmonary artery banding at the age of 2 months. After this procedure, residual pulmonary venous stenosis was noted, and reoperation for pulmonary vein stenosis release and pulmonary artery re-banding was planned at 9 months of age. At that time, CT revealed pectus excavatum and hypertrophic retrosternal tissue, but no cardiac compression was observed because of dextrocardia (**Fig. 1**).

**Fig. 2:**
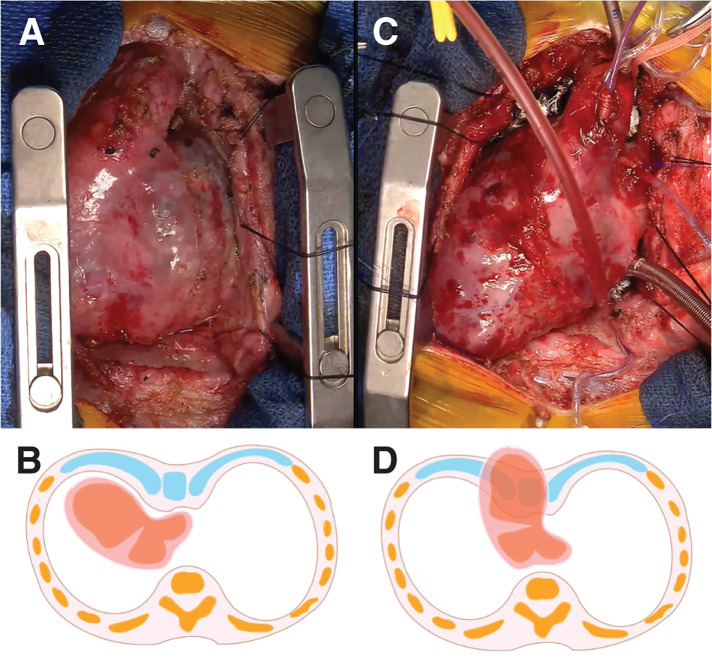
First operation: sutureless repair for postoperative pulmonary venous obstruction. (**A**) Preoperative condition showing pectus excavatum with dextrocardia. (**B**) Schematic illustration corresponding to (**A**). (**C**) Post-cardiac surgery condition demonstrating anterior displacement and protrusion of the cardiac apex, resulting in failure of chest closure; only temporary skin closure was performed (mesocardia). (**D**) Schematic illustration corresponding to (**C**).

Since the 1st surgery, progressive pectus excavatum had been observed with initial rightward cardiac rotation sparing the heart from direct compression (**Fig. 2A** and **2B**). At reoperation, after mediastinal dissection and correction of rotation, the heart returned to a more central position and was directly compressed between the sternum and deformed costal cartilages. This patient’s complex cardiac anatomy—right atrial isomerism with single atrium and single ventricle—was predisposed to marked mediastinal mobility. During the 2nd operation, extensive pericardial and pulmonary venous dissection corrected the previous rightward cardiac rotation (dextrocardia) and brought the heart into a midline position (mesocardia-like). This positional change, combined with severe pectus excavatum and hypertrophic retrosternal tissue, resulted in acute mediastinal compression and functional cardiac tamponade, primarily affecting the right heart system during attempted chest closure. Attempts at conventional closure produced abrupt increases in central venous pressure and severe systemic hypotension, confirming mechanical compression of the heart (**Fig. 2C** and **2D**). At the end of pulmonary venous stenosis release, central venous pressure increased from 5 to over 14 mmHg upon attempted closure, with blood pressure dropping by more than 15 mmHg and narrowing of pulse pressure. Only skin closure was performed, leaving the sternum and mediastinum open, and the patient was returned to the ICU with hemodynamic stabilization under volume optimization and vasoactive support.

**Fig. 3:**
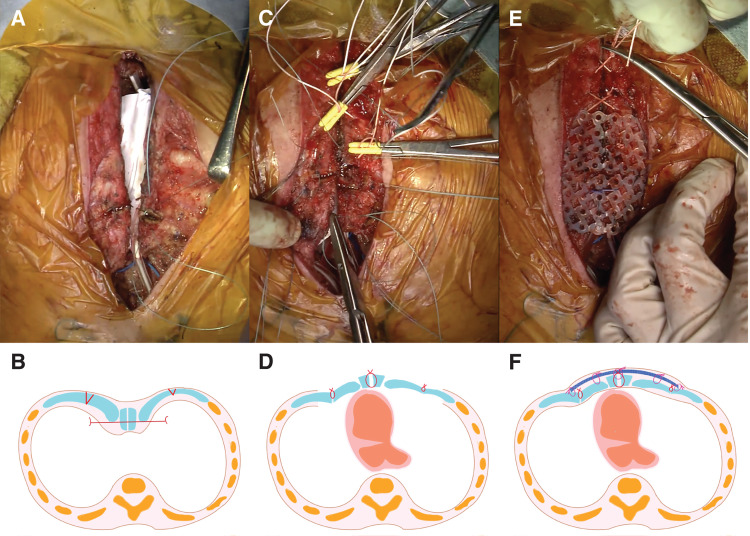
Second operation: thoracic reconstruction. (**A**) Resection of costal cartilages, transverse sternal osteotomy, and removal of hypertrophic retrosternal tissue. (**B**) Schematic illustration corresponding to (**A**). (**C**) Suturing of costal cartilages and the sternum following remodeling. (**D**) Schematic illustration corresponding to (**C**). (**E**) External fixation using a bioabsorbable PLLA mesh plate (Grand Fix). (**F**) Schematic illustration corresponding to (**E**). Grand Fix, Gunze Limited, Kyoto, Japan; PLLA, poly-L-lactic acid

Given the infant’s age and ongoing somatic growth, the surgical team avoided permanent prostheses or rigid materials beneath the sternum that could predispose to infection or growth restriction. Instead, they selected an external fixation method based on the Rehbein technique using a bioabsorbable PLLA mesh plate (Grand Fix; Gunze Limited, Kyoto, Japan) that provides structural support for approximately 6 months before gradual resorption. Through the existing median sternotomy incision, subperichondrial dissection exposed the anterior aspects of the costal cartilages with trimming of hypertrophic portions and wedge resections at the sternocostal junctions (**Fig. 3A** and **3B**) and onset of concavity to redirect the chest wall anteriorly (**Fig. 3C** and **3D**). The PLLA plate was softened in 70°C saline, molded into a convex contour to simulate pectus carinatum, and placed anterior to the sternum secured with 3–0 nonabsorbable sutures (**Fig. 3E** and **3F**). This effectively elevated the sternum, expanded the mediastinum, and prevented compression (**Supplementary Video**). Trial closure showed stable hemodynamics with central venous pressure around 8 mmHg. After placement of bilateral Blake drains and layered closure of muscle and skin, the patient stabilized but developed right phrenic nerve palsy likely related to dissection. This required CPAP for 3 weeks before gradual recovery permitted weaning.

**Fig. 4:**
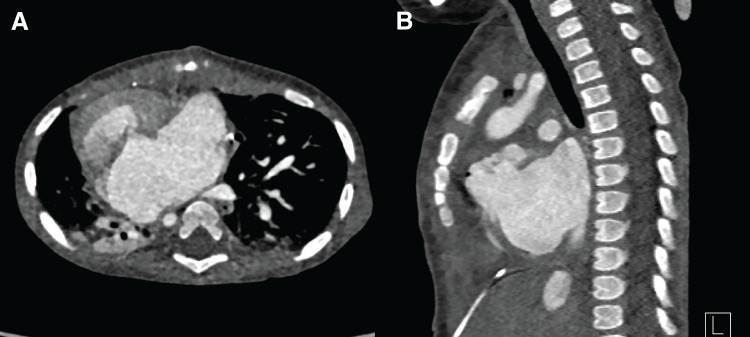
Postoperative evaluation. (**A**) Axial CT image demonstrating cardiomegaly without evidence of external sternal compression. (**B**) Sagittal CT view demonstrating adequate sternal elevation and improved mediastinal volume.

Low-dose heparin was administered to reduce risk of pulmonary venous thrombosis. Postoperative CT demonstrated improved thoracic contour (**Fig. 4**). The patient was ultimately discharged 2 months after surgery in good condition with stable respiration and appropriate growth.

## DISCUSSION

PE is the most prevalent congenital deformity of the anterior chest wall, but its clinical implications in infancy are seldom emphasized. While the majority of cases are detected during early childhood and progress gradually with growth, severe forms of PE may exert hemodynamic consequences even in neonates and infants, particularly in those with underlying CHD. In the present case, PE directly interfered with postoperative chest closure following reoperation for pulmonary venous stenosis in a 9-month-old boy with complex CHD. This scenario exemplifies a unique intersection of cardiac surgery, thoracic deformity, and innovative reconstructive techniques aimed at preserving growth potential. The following discussion places our case in the context of existing literature, emphasizing the pathophysiology of PE, its relationship to CHD, prior surgical strategies in pediatric patients, and the specific advantages of bioabsorbable materials such as PLLA mesh plates.

### Pathophysiology of pectus excavatum and cardiac implications

PE is characterized by posterior displacement of the sternum and costal cartilages, resulting in a depressed anterior chest wall.^1)^ Although cosmetic concerns often dominate clinical attention, significant deformities can impose mechanical effects on the heart and lungs. In adolescents and adults, cardiopulmonary exercise testing frequently reveals reduced aerobic capacity and ventilatory efficiency.^5)^ Importantly, in infants, the smaller mediastinal volume and relatively larger cardiac size magnify the potential impact of PE. Sonaglioni et al. demonstrated that chest shape influences ventricular–arterial coupling parameters in infants with PE, providing evidence that even subtle deformities may alter cardiac mechanics.^3)^

In rare but dramatic cases, PE can cause direct obstruction of cardiac inflow or outflow. Borrhomée et al. reported a case of syncope resulting from right ventricular obstruction caused by severe PE.^2)^ Such reports highlight that beyond cosmetic deformity, PE may pose life-threatening risks. Our patient presented a similar pathophysiological mechanism: once the heart rotated centrally after pulmonary venous repair, the sternum and deformed costal cartilages compressed the enlarged right heart, resulting in a sharp increase in CVP and systemic hypotension during attempted closure. This underlines the hemodynamic fragility of infants with concurrent PE and CHD.

### Relationship between pectus excavatum and congenital heart disease

The association between chest wall deformities and CHD has long been recognized. Shamberger in a seminal report, demonstrated that anterior chest wall deformities are more common in patients with CHD compared with the general population.^9)^ The mechanisms underlying this association remain speculative but may include shared developmental pathways, mechanical factors from repeated sternotomies, and postoperative changes in thoracic growth dynamics. Park and Varma also emphasized that PE may serve as a diagnostic clue in children with mitral valve prolapse, reinforcing the concept that chest wall deformities and cardiac abnormalities may coexist more often than previously appreciated.^4)^ In our patient, PE developed after the initial sternotomy for TAPVC repair, consistent with the notion that repeated thoracic surgery can exacerbate or even precipitate chest wall deformities.^9)^ This is particularly relevant in neonates and infants, whose costal cartilage is highly malleable. The interplay between mediastinal scarring, altered respiratory mechanics, and sternal instability may accelerate deformity formation. Thus, pediatric surgeons and cardiologists must be vigilant in recognizing the potential for PE to become not only a cosmetic but also a functional problem in this population.

### Surgical strategies for pectus excavatum in infants and children

Historically, surgical correction of PE was reserved for older children and adolescents. Shamberger and Welch provided one of the earliest systematic evaluations of surgical repair in pediatric populations, establishing the foundation for open techniques such as the Ravitch procedure.^6)^ Over time, minimally invasive methods, particularly the Nuss procedure, have become predominant, with large series confirming their effectiveness across different age groups.^7)^ Nevertheless, the optimal timing of repair remains debated. While earlier intervention may prevent progression and reduce psychological burden, concerns persist about recurrence and interference with thoracic growth when surgery is performed too early.^1,6)^ In infants, surgical repair of PE is extremely rare. Takabayashi et al. described a unique case of suture repair performed simultaneously with cardiac surgery in an infant.^13)^ Their report highlights that in select circumstances, early intervention may be necessary to ensure adequate mediastinal space. Similarly, Wang et al. demonstrated the feasibility of simultaneous repair of CHD and PE in young children, achieving satisfactory outcomes without significant complications.^12)^ Rousse et al. described staged repair of PE during an aortic valve-sparing operation, further supporting the concept that thoracic deformity correction can be integrated into cardiac surgical strategies.^11)^ Yeung et al. also reported a case of severe asymmetric PE complicating aortic repair in a patient with Marfan’s syndrome, illustrating the diverse contexts in which thoracic deformities can interfere with cardiac interventions.^15)^ Despite these encouraging reports, most authors agree that PE repair in infants should be approached with caution. The high plasticity of the thoracic cage increases the risk of recurrence, while the presence of concomitant CHD complicates postoperative management.^10,14)^ Nonetheless, in life-threatening scenarios where PE prevents chest closure, immediate surgical innovation becomes mandatory.

### Risks and outcomes of minimally invasive approaches after CHD surgery

Minimally invasive techniques, particularly the Nuss procedure, have been widely applied in pediatric populations with and without CHD. Chen et al. evaluated different Nuss procedures for PE in patients after CHD surgery and emphasized the need for careful risk management due to adhesions and altered anatomy.^14)^ Similarly, Li et al. reported their institutional experience with Nuss repair following CHD surgery, confirming feasibility but cautioning about increased technical complexity.^10)^ These studies highlight that prior cardiac surgery poses additional risks for PE repair, including injury to great vessels and arrhythmias. In infants, these risks are magnified. The small thoracic cavity, friable tissues, and limited cardiopulmonary reserve make minimally invasive repair technically challenging. In our case, a conventional Nuss approach was not feasible. Instead, a modified Rehbein strategy using an external fixation device was employed, adapted to the unique anatomical and physiological needs of the patient.

### Advantages of bioabsorbable materials in chest wall reconstruction

One of the most distinctive features of this case was the use of a bioabsorbable PLLA mesh plate. Traditional rigid prosthetic implants, such as titanium bars, offer durable structural support but carry the risk of infection and long-term interference with thoracic growth. In contrast, PLLA plates gradually degrade over approximately 6 months, providing temporary stabilization during critical periods of healing and remodeling. This property is particularly advantageous in infants, where long-term growth must remain unimpeded. Furthermore, PLLA plates can be molded intraoperatively in warm saline to achieve the desired contour. The PLLA mesh plate was temporarily softened in 70°C saline for molding. This standard thermoforming process allows intraoperative contouring without impairing mechanical strength or biocompatibility, as the plate regains its rigidity upon cooling and maintains stability until bioresorption. Bioabsorbable PLLA devices have been widely used for sternal and chest wall stabilization in cardiovascular and thoracic surgery. Early reports demonstrated the safety and feasibility of PLLA sternal pins after median sternotomy, while subsequent biomechanical and clinical studies showed that bioresorbable osteosynthesis materials promote stable bone healing and early sternal fusion.^16–18)^ Pediatric studies also indicated that resorbable fixation devices enhance sternal stability without interfering with growth.^19)^ A recent randomized trial confirmed that PLLA sternal pins provide superior postoperative stability and less pain compared with conventional wire closure.^20)^ In this context, our use of a bioabsorbable PLLA mesh plate as an external fixation method extends previous experience with pin-based reinforcement to a growth-sparing reconstruction strategy in an infant where primary chest closure was not feasible. In our patient, the plate was shaped to simulate a pectus carinatum-like configuration, thereby increasing mediastinal volume and relieving cardiac compression. Importantly, external placement of the bioabsorbable plate helped avoid postoperative mediastinitis, one of the most severe complications following repeated sternotomy. By positioning the device anterior to the sternum, no foreign material was introduced into the mediastinum, thereby preventing exposure of prosthetic material to the previously dissected and potentially colonized retrosternal space. Moreover, this technique preserved sternal perfusion and allowed complete soft-tissue coverage, further reducing the likelihood of deep sternal infection. This strategy resonates with broader trends in thoracic surgery, where absorbable materials are increasingly applied to balance immediate structural needs with long-term growth considerations. The innovative application of such materials in an infant underscores their potential utility in salvage scenarios where conventional techniques are either unsafe or impractical.

### Lessons learned and future directions

Our case highlights several key lessons. First, PE in infants with CHD is not merely a cosmetic issue but can become a critical determinant of surgical feasibility and postoperative stability. Clinicians should maintain a high index of suspicion for the functional impact of PE in this population, particularly when planning reoperations. Second, surgical innovation tailored to growth considerations is essential. While minimally invasive repairs dominate in older children, alternative techniques such as bioabsorbable external fixation may be more appropriate in infants. Third, long-term follow-up will be necessary to assess outcomes. Although the immediate hemodynamic results were favorable, questions remain about the durability of correction after PLLA degradation, the risk of recurrent deformity, and the long-term cardiopulmonary implications. The literature suggests that early repair may reduce some functional limitations, yet recurrence remains a significant concern.^1,6)^ Whether temporary external fixation can alter long-term outcomes in infants is unknown. Larger series and multicenter collaborations will be required to establish standardized approaches. Nonetheless, our case adds to the growing body of evidence that PE repair can and sometimes must be undertaken in infancy when dictated by life-threatening circumstances.

## CONCLUSIONS

This case demonstrates that severe PE in an infant with complex CHD can preclude conventional chest closure and necessitate innovative reconstructive strategies. The use of a bioabsorbable PLLA mesh plate provided effective temporary external fixation, allowing safe closure without compromising growth potential. This approach represents a valuable addition to the surgical armamentarium for managing rare but critical situations in pediatric cardiac surgery. As the literature on PE and CHD continues to expand, our experience emphasizes the need for individualized, growth-sparing interventions that prioritize both immediate survival and long-term development.

## SUPPLEMENTARY MATERIALS

Supplementary Video Intraoperative video of thoracic reconstruction using a poly-L-lactic acid mesh plate.

## DECLARATIONS

### Availability of data and materials

The data that support the findings of this study are available on request from the corresponding author. The data are not publicly available due to privacy or ethical restrictions.
